# Medical Electricity

**Published:** 1881-12

**Authors:** William J. Morton

**Affiliations:** 15 East 45th St., New York


### ARTICLE XII.

TO THE EDITORS OF THE CHICAGO MEDICAL JOURNAL AND EXAMINER :—I have read with pleasure and profit Dr. Clevenger's article on Medical Electricity in the November number of the MEDICAL JOURNAL AND EXAMINER, and will say at once that I am in hearty accord with his criticisms upon inexact, unscientific and pretentious electro-therapeutic measures. This protest against indiscriminate electrization should be entered again and again until no reputable physician would dare to pump, so to speak, electricity into or through his patient with the view of counteracting a suspected, though unlocalized and undiagnosticated diseased condition. I have always contended that the conscientious practice of electrization must be founded on the dictum of Benedict, viz : To treat the seat of disease, or, to make an amendment to this rule often found necessary, to treat also the part affected. For instance, in a hemiplegia both cerebrum and the affected nerves and muscles are to be treated. Regarding, then, both an accurate diagnosis and a localized treatment as essential, I can but sympathize with Dr. Clevenger's criticisms upon unscientific methods. The terms "central galvanization," and "general faradization," for instance, appear to me to be as pernicious as unscientific. But, just at this point, and in this connection, I wish to say that I fear Dr. Clevenger has received a wrong impression of the methods pursued by Charcot and Vigouroux in statical electrizations. Referring to a discussion at the meeting of the American Neurological Association held in June last, he writes : "The methods of Charcot and Vigouroux in the use of a large platform whereon numbers of patients were electrized at once, were characterized as unwarrantable." To be sure, he was justified in this idea by the words of a member * of the Association, who stated that " they " (Charcot and Vigouroux) would huddle together upon a single isolated stool cases of hystero-epilepsy, locomotor ataxia, paralysis agitans, a case of anæsthesia, and a case of headache, thus forming a series of pathological conditions which had nothing in common, and apply the same current to all of them." The term " unwarrantable " should rather be applied to this last quoted statement than to Charcot's methods. The idea conveyed is entirely fallacious. The treatment did not consist in being " huddled " upon a platform to which static electricity was conveyed. This was merely an essential preparatory condition of general " electrification " or " charge." The real treatment consisted in drawing sparks from the seat of disease in each instance where this was practicable—in the ataxic, from over the spine, in paralysis agitans from over the spine and from the shaking members, in the hystero-epileptic, and the anæsthetic from the affected part, and in cases of peripheral paralysis from the nerve involved. Surely this was conscientious and scientific electrization, and scarcely deserves to be called an unwarrantable method ; unless, indeed, it is " unwarrantable " to try new remedies, or methods, or forms of electricity ; unless, indeed, it is more desirable to confine oneself to what each critic may consider the settled and fixed facts of medicine, and to assert that there is no opportunity for advance in the treatment of disease.

But I do not here intend to re-argue the claims that static electrization has upon the medical profession. These claims I have already set forth in several publications, notably in a paper read before the New York Academy of Medicine, March 3, 1881, and printed in the *New York Medical Record* of April 2, 1881.

In this paper I first led the way to the present resurrection of statical electrization in America, following the indications and teachings of Charcot and Vigouroux in France. I am gratified that the subject is receiving a fair investigation.

From the standpoint of the medical electrician I have little

* Dr. Amidon.

interest in the matter, though I think that in the future the specialist in electricity will feel obliged to resort to statical as well as galvanic and faradic electrizations ; but as a physician interested in the neurological side of diseases, I am confident that statical electrization has a place in effective treatment as fixed, understandable and reliable as strychnia, the bromides, the douche, or as have the more familiar galvanization and faradi zation.

Waiving, then, discussion as to the present status of statical electricity, I ask brief space for comment on a point more nearly personal.

Referring still to the last meeting of the American Neurological Association, Dr. Clevenger remarks: "Dr. Morton described an apparatus, *by means of which he claimed results similar to those obtained by the secondary coil, and erroneously called it a new induction current.*" It is not probably meant that I called the "apparatus" a new "induction current;" but rather that I claimed to have produced results similar to those obtained by the secondary coil, and that I claimed that I produced these results by means of an induction current, and that this induction current was new.

And first in regard to the results:

The results referred to are nerve and muscle reactions, produced from a Holtz machine, and exactly similar to those produced by the ordinary Faradic machines. These results were publicly and practically demonstrated before the New York Academy of Medicine, and they have been admitted by all physicians and electrical experts who have witnessed them.

It is of these reactions that Dr. Bartholow writes: "The impression made by the electricity is like that of the faradic machine, but is much less painful, and strong muscular contractions are thus induced with greatly less pain than can correspondingly strong contractions of the muscles be obtained by faradism. We have thus added to our resources an immensely useful instrument for the production of these effects hitherto obtained from faradism."

Dr. Bartholow's observations have all the more force, since they were made independently of my own experiments, and were published only a few weeks later.

That I justly "claimed" to have produced the results in question may, then, be considered as settled.

Next, as to the second point, that I "erroneously" called the current "a new induction current." Now in all friendliness, I ask Dr. Clevenger to substantiate this criticism. I shall be as glad as any one to be set right in the matter. It is a question not without interest to electricians, owing to the wide range of usefulness possessed by induced currents, and this one in particular, in helping to restore paralyzed muscles and degenerated nerves to their normal functions.

I wish to demonstrate to him, respecting as I do his criticism, and the pages of the journal whence it proceeds, first, that the current I described is a true induction current; and second, that it is new. This demonstration, I may add, seems to me to be superfluous to any one who has evoked the current in actual practice from a Holtz machine. A moment's inspection by any one familiar with induced currents would save pages of writing. And I would apply the same remark to my critics * in the American Neurological Association, who seem to have entirely misapprehended the question. I can only hope that Dr. Clevenger has prematurely accepted their criticism as final, without personal investigation and knowledge of what I claim.

To produce the current in question, remove the connecting bar or chain between the two *outer* tin-foil coatings of the two Leyden jars usually attached to a Holtz machine. Connect ordinary conducting wires and wet-sponge electrodes to each outer coating respectively, and finally connect the two *inner* coatings by the discharging rod. As soon as the machine is set in motion, and the Leyden jars are filled, the discharging rods must be drawn out a very small fraction of an inch, and at once a current is felt between the two sponge electrodes, which in its general characteristics cannot be distinguished from the ordinary Faradic current. The experiment succeeds best with a large Holtz machine.

Next, is this an induced current?

It is a "current," according to the recognized definitions of

* Drs. Amidon, Gradle and Birdsall.

the term, for it is an equalization of two potentials along a conductor. Every electric discharge, however brief, is a current; the spark is a brief current. The voltaic, or so-called "continued current" is no more nor less than a succession of brief discharges; in other words, the potentials are continuously kept up to a rapid discharging point. The Faradic, or induced current, in the same manner, consists simply of the alternations of direction in a current set up through a dielectric by a voltaic discharge; *i. e.*, a "make" and "break" in the battery current.

I consider it demonstrated, then, that whatever electrical process takes place along the conducting wires or through the wet-sponge electrodes, while a Holtz machine is arranged and working as above described, is a current. For the unequal potentials exist in the *outer* tinfoils of the Leyden jars, constantly kept at a fixed point of discharge by the working of the machine; while their equalization takes place along the conducting wire, and between the electrodes properly placed. In this current, as in the induced current, the term is loosely applied to a succession of alternating discharges or influences along a conductor.

Next, is this current an induced current?

It is *induced* or created by influence through the dielectric glass, and by the respective positive and negative electricities *inside* the jars.

We will, for instance, set the machine in motion with its discharging rods one-sixteentn of an inch apart. What happens? A spark, *i. e.*, a current in brief form, passes between the positive electricity of the inside of one jar and the negative electricity of the inside of the second jar. At the same instant, an induced current, *i. e.*, a brief equalization of potentials, takes place between the outsides of the same two Leyden jars. If, now, the discharges from the inside of the two jars are very rapid, as between the electrodes of a Holtz machine, the discharges on the outside are also very rapid, but the discharges from the inside, passing through a bad conductor like air, take the form of zigzag lines of so-called sparks; while the discharges from the outside, being led through tolerably good conductors, do not take a spark form (though they may be made to do so), and we thus have at the sponge electrodes connected to the two respective outsides an "induced current." It is "induced" for the reasons given. It is a current for the reason given. It is rapidly alternating, like any true induced current; and, to carry the analogy further, the discharge between the inside of the jars is no more nor less than the counterpart of the "battery current," and the succession of spark discharges at the usual discharging rods is no more nor less than the ordinary "make" and "break" in the battery current of an ordinary Faradaic machine. In the one case a continued static discharge, or equalization of potentials, takes place; in the other, we have a voltaic or galvanic discharge and equalization of potentials.

Now, the current obtained from any induction coil (ordinary Faradaic batteries) is often termed the Voltaic induced current. I ventured to call, by analogy, the current I have discovered the "static induced." I have yet to be shown that my terminology is not well selected.

Lastly, as to the point: Is this induction current new?

As a current capable of producing muscle and nerve reactions similar to other induction currents, I claim that it is. Its novelty lies not in the simple scientific fact that a discharge of static electricity provokes in a neighboring but untouching conductor an induced discharge or current, but in the fact that this induced discharge or current of static electricity may be so affected by an *interruption* in the inducing discharge as to cause physiological action in nerve and muscle. Uninterrupted, this induced current produces in the human body no appreciable effect—interrupted, it is a serviceable ally in medicine. After many attempts at manufacturing an efficient interrupter, I found that the spark discharge between the discharging rods was the simplest and best.

And medically it is "a new induction current." In spite of the large medical use to which statical electricity has been put during more than a hundred years, I am not aware that any one, before my experiments or publication, made use of any similar medical application of static electricity by means of any arrangement of Leyden jars with any form of frictional or inductive electric machine. I may then, in short, say that although an induction discharge of static electricity was known to exist, no one ever interrupted it and applied it to the human body, and got physiological results. Herein lies its novelty, and herein it is a discovery or invention.

This is not the place to refer to the physiological effects of the new current. I will simply say that it seems to me, and to others who, on investigation, have come to the same conclusion, that the static induced current produces stronger and more diffused muscular contractions, and with less pain, where pain is necessarily produced, than the voltaic induced currents now in use. In many cases of paralysis (of the anterior tibial group of muscles, of the deltoid, in Bell's paralysis, in infantile spinal paralysis, etc., etc.) I have obtained muscular contractions, where I have not been able to do so with a Gaiffe battery, or with the battery manufactured by the New York Galvano-Faradic company.

I am quite ready to admit that I "must experiment and report more fully before the apparatus will win favor." This I expect to do; but in the meantime, I am sure Dr. Clevenger and the JOURNAL AND EXAMINER will appreciate my desire to make clear to them the fundamental facts in the matter, even though it require what I fear is a somewhat lengthy letter, to arrive at the result. I am

Very respectfully yours,

WILLIAM J. MORTON.

15 EAST 45TH ST., NEW YORK, Nov. 26, 1881.

---

MEN OF PROGRESS.—There is soon to be published a work with this title. The publisher calls upon the physician, who dictates his own life; he next pays for an engraving of himself, and presto becomes great. It is difficult to escape such a fate. —*Medical News.*

